# Ability of emergency physicians to diagnose acute coronary syndrome on the ECG of acute chest pain patients

**DOI:** 10.1186/1757-7241-23-S1-A20

**Published:** 2015-07-16

**Authors:** Catarina E Hilmersson, Jonas Björk, Mattias Ohlsson, Ulf Ekelund

**Affiliations:** 1 Department of Emergency Medicine, Skåne University Hospital, Lund, Sweden; 2 Region Skåne Competence Centre for Clinical Research, Lund, Sweden; 3 Computational Biology and Biological Physics Group, Lund University, Lund, Sweden

## Background

Interpretation of the ECG is central to the diagnosis of acute coronary syndrome (ACS) in emergency department (ED) chest pain patients. Failure to recognize ECG signs of cardiac ischemia is a common cause of missed ACS. This study aimed to investigate ED doctors' ability to diagnose ACS based solely on ECGs from ED chest pain patients.

## Methods

80 male and female ED physicians each received 20 ECGs and answered two questions for each ECG: #1 "Will this patient's discharge diagnosis be ACS?" (yes/no), and #2 "On a scale of 0-100, how likely is it that the patient will be discharged with an ACS diagnosis?" Data on each physician's gender, experience, and daily practice in ECG reading were collected. Physicians' answers were assessed for correctness when compared to discharge diagnosis and expert ECG interpretation, respectively.

## Results

The physicians' sensitivity and specificity for ACS were 67% and 75% when compared with discharge diagnosis, and 100% and 74% when compared with expert ECG interpretation. Female physicians showed a significantly higher sensitivity for ACS than male physicians (medians 75 vs. 50%; p = 0.038) when compared with discharge diagnosis, but not with expert interpretation. On a scale of 0-100, female physicians assigned a higher ACS likelihood than male physicians irrespective of the patient's discharge diagnosis, and also in patients the physician did not believe to have ACS. ROC-area was not significantly different for male and female physicians. There was no difference in diagnostic ability based on work experience and number of ECGs read daily.

## Conclusion

Female ED physicians correctly identified more patients who were discharged with ACS than their male colleagues, probably explained by a tendency to assign a higher likelihood of ACS. ED physicians' ECG interpretation correlated better with expert interpretation than discharge diagnosis, which indicates that even a well interpreted ECG cannot reliably predict an ACS discharge diagnosis. There was no gender difference in the overall ability to diagnose ACS on the ECG, and further studies are needed to elucidate if gender differences in ECG interpretation translate into differences in clinical decision-making.

